# New species of parasitic nasal mites infesting birds in Manitoba, Canada (Mesostigmata, Rhinonyssidae)

**DOI:** 10.3897/zookeys.786.28767

**Published:** 2018-09-25

**Authors:** Wayne Knee

**Affiliations:** 1 Canadian National Collection of Insects, Arachnids, and Nematodes, Agriculture and Agri-Food Canada, 960 Carling Avenue, K.W. Neatby Building, Ottawa, Ontario, K1A 0C6, Canada Agriculture and Agri-Food Canada Ottawa Canada

**Keywords:** Acari, alpha taxonomy, mite, nasal mite, parasite

## Abstract

Mites (Acari, Acariformes, Parasitiformes) are one of the most diverse and abundant groups of arthropods associated with birds. Several families of mites have colonised the respiratory tract of birds, the Rhinonyssidae (Mesostigmata) being the most diverse. There are 66 species of rhinonyssids (59 named, seven undescribed species) reported from Canadian birds. Two new species of rhinonyssids were discovered while surveying nasal mites from birds in Manitoba, Canada, and are herein described as *Sternostomagallowayi***sp. n.** from the horned lark (*Eremophilaalpestris*), and *Vitznyssuserici***sp. n.** from the common nighthawk (*Chordeilesminor*).

## Introduction

Birds are hosts to a hyperdiverse assemblage of symbiotic animals, residing in all conceivable habitats. Mites are one of the most diverse and frequently encountered associates of birds, with at least 40 families and 2500 described species that live in close association with birds (Proctor and Owens 2000). The symbiotic relationships between birds and their associated mites are varied. Many species are relatively benign such as most feather mites (Astigmata: Analgoidea, Pterolichoidea), while other species are deleterious parasites, such as the northern fowl mite (*Ornithonyssussylviarum* (Canestrini and Fanzago) Mesostigmata: Macronyssidae) or scaly-leg mites (*Knemidokoptes*, Prostigmata: Knemidokoptidae) (Fain and Elsen 1967, Proctor and Owens 2000, Mullens et al. 2009).

Mites inhabit all parts of the avian integument; they can be found on and in the skin of their hosts, on and in feathers, and in the respiratory tract. Five families of acariform (Cloacaridae, Ereynetidae, Turbinoptidae, Cytoditidae) and parasitiform (Rhinonyssidae) mites, have evolved independently to be parasitic in the nasal passages or lungs of birds. There are at least 500 described species of nasal mites worldwide (Fain 1994), of which the Rhinonyssidae are the most diverse and abundant, with 66 species (59 named, 7 undescribed species) occurring in Canada (Knee and Galloway 2017). Rhinonyssids are slow-moving, obligate haematophagous endoparasites that dwell primarily in the nasal cavity and turbinates, though occasionally these mites invade the trachea, lungs and body cavity (Porter and Strandtmann 1952, Bell 1996). Typically, rhinonyssids are not considered to cause significant damage to their hosts; however, the feeding activity may cause trauma to nasal tissues (de Rojas et al. 2002), and species that invade the lungs and air-sacs, such as *Sternostomatracheacolum* Lawrence, can cause pneumonia and death of their hosts (Bell 1996).

Nasal mites have been surveyed in many geographic regions throughout the world (Fain 1957a, Maa and Kuo 1965, Domrow 1969, Pence 1975, Butenko 1984, Knee and Galloway 2017); however, despite these efforts, our understanding of this mite assemblage is largely incomplete. A recent survey of nasal mites (Rhinonyssidae, Turbinoptidae, Ereynetidae) infesting birds in Manitoba (Knee and Galloway 2017) uncovered two new species of rhinonyssids. Herein, I propose and describe *Sternostomagallowayi* sp. n., and *Vitznyssuserici* sp. n.

## Materials and methods

Injured and sick birds were submitted to the Wildlife Haven (Manitoba Wildlife Rehabilitation Organization) and the Prairie Wildlife Rehabilitation Centre, mostly by members of the public, and after death they were stored at -20 °C until processing in TD Galloway’s lab at the University of Manitoba (Winnipeg, Manitoba, Canada). The nasal passages of thawed birds were flushed with a solution of warm water and mild soap using a 12 mL Monojet® 412 curved tip orthodontic plastic syringe. The solution was flushed through each nostril, through the opening in the palette and back out the mouth into a Petri dish. Occasionally nasal mites were collected from whole-body washing, where a thawed bird was placed in a container ranging in volume from 4–40 L, depending on the size of the bird, submerged in warm water containing a few drops of liquid dish detergent. Each bird was agitated vigorously three to 10 minutes, depending on the size of the bird. Each bird was then removed from the container and rinsed thoroughly; the washing solution was filtered through a 90 µm sieve. This process was repeated once again with warm, soapy water, and once finally with warm water. The filtrate from all three washes was preserved in 70 or 95% ethanol. Samples were examined for mites using a dissecting microscope. All nasal mites were collected and preserved in 70 or 95% ethanol for later identification.

Mites were removed from ethanol and cleared in 85% lactic acid, mounted in polyvinyl alcohol medium (6371A, BioQuip Products, Rancho Dominguez, California, United States of America), and cured on a slide warmer at 40 °C for 3–4 days. Slide-mounted specimens were examined using a Leica DM2500 compound microscope and Leica ICC550 HD camera, with differential interference contrast illumination (DIC). Initial drawings of mites were made with pencil on paper using a camera lucida. Illustrations were later merged in Adobe Photoshop CS5 and redrawn in Adobe Illustrator CS5 using an Intuos 3 Graphics Tablet from WACOM Co., Ltd. (Saitama, Japan). Leg chaetotaxy is based on the system proposed by Evans (1963) and Evans and Till (1965). Idiosomal chaetotaxy follows the system of Lindquist and Evans (1965). All measurements are in micrometres (µm), and measurements presented with the mean followed by the range in parentheses. Rhinonyssids have a reduced setal compliment when compared to most free-living Mesostigmata, and they frequently have unpaired setae on the idiosoma and legs; therefore, the number of setae on the legs and idiosoma can be variable between specimens within a species.

Type specimens are deposited in the Canadian National Collection of Insects, Arachnids, and Nematodes (Agriculture and Agri-Food Canada, Ottawa, Ontario, Canada). Host taxonomy follows Chesser et al. (2018)*Checklist of North American birds*, 7^th^ edition, plus supplements. Scientific permits were issued to TD Galloway for migratory birds by the Canadian Wildlife Service, Environment Canada.

## Results and discussion

### Family Rhinonyssidae Trouessart, 1895

#### Genus Sternostoma

Taxon classificationAnimaliaMesostigmataRhinonyssidae

Berlese & Trouessart, 1889

##### Type species.

*Sternostomacryptorhynchum* Berlese & Trouessart, 1889.

**Diagnosis.** Stigmata dorsal or lateral, without peritreme. Gnathosoma ventral, only partially visible dorsally. Cheliceral digits small, less than one tenth the length of the chelicerae. One or two dorsal plates. Sternal and anal plates usually present.

#### Sternostoma gallowayi sp. n.

Taxon classificationAnimaliaMesostigmataRhinonyssidae

http://zoobank.org/09B2A5E1-AB6E-42DB-9302-12A731B55EEE

**Figure 1.**
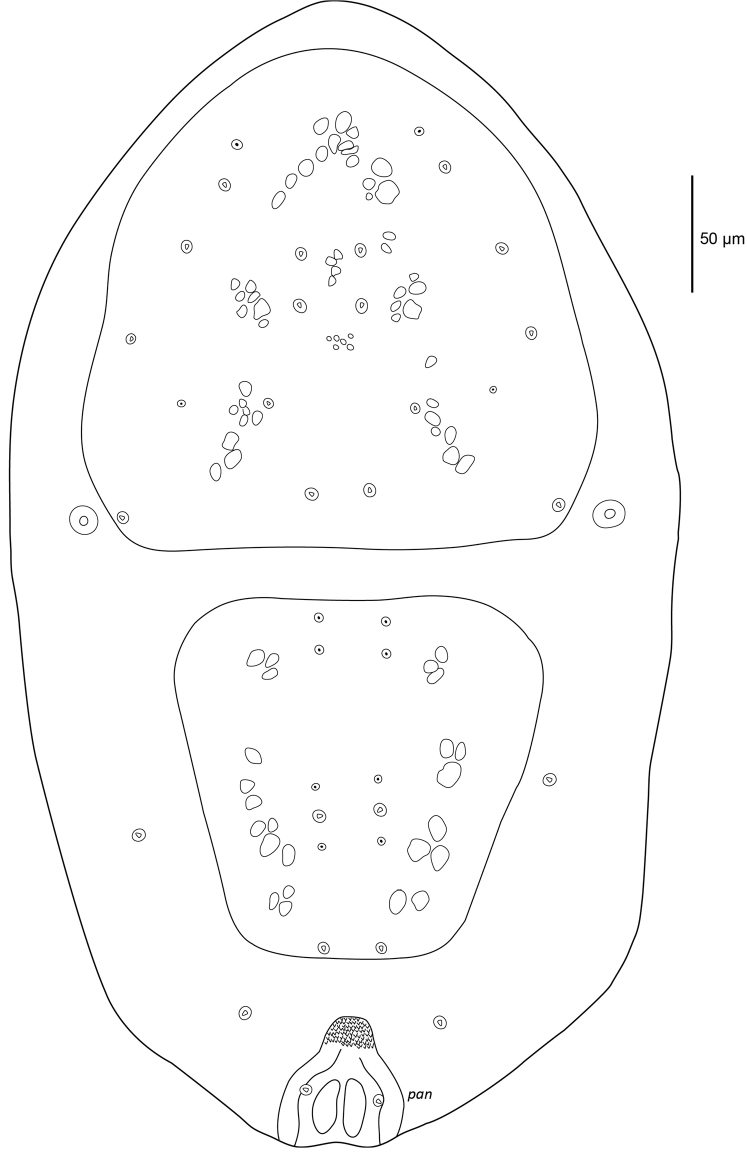
Female *Sternostomagallowayi* sp. n. dorsal idiosoma.

**Figure 2.**
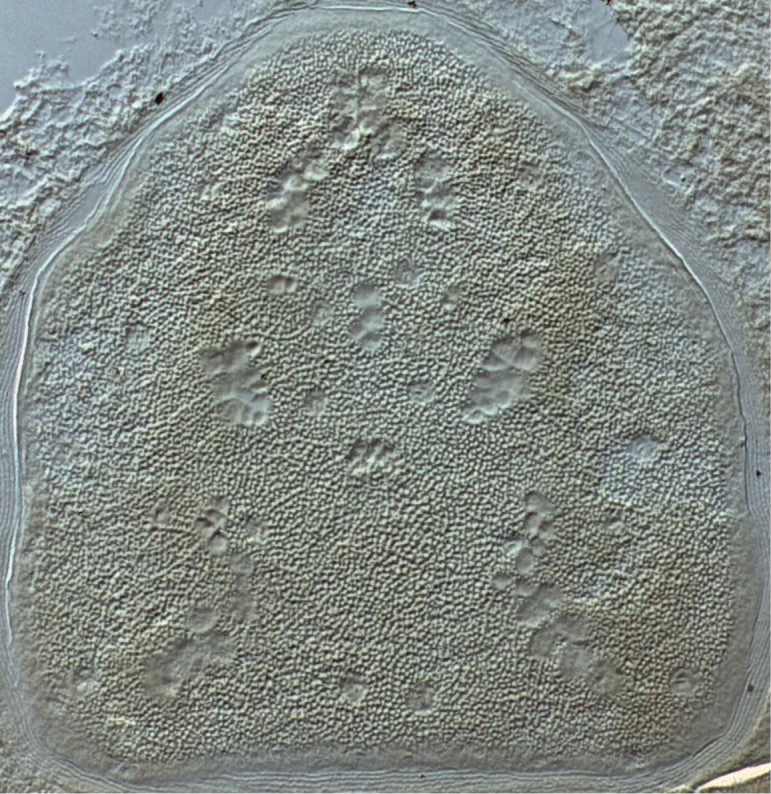
Female *Sternostomagallowayi* sp. n. podosomal shield.

**Figure 3.**
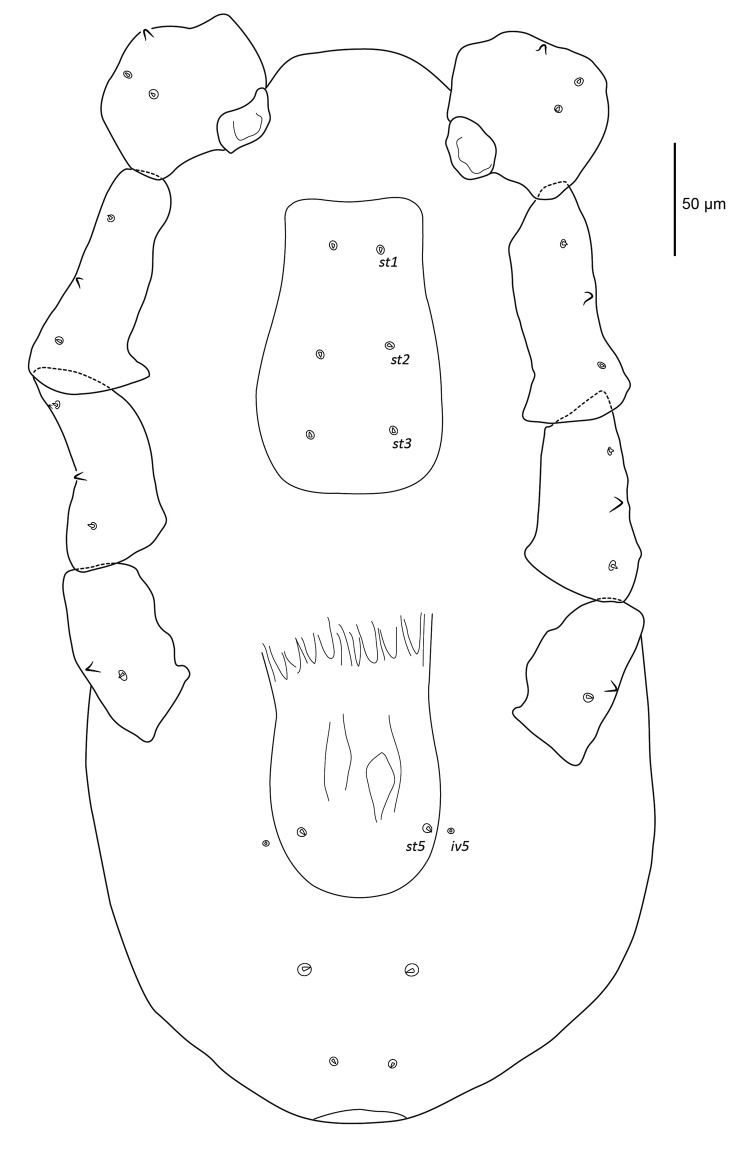
Female *Sternostomagallowayi* sp. n. ventral idiosoma including coxae.

**Figure 4.**
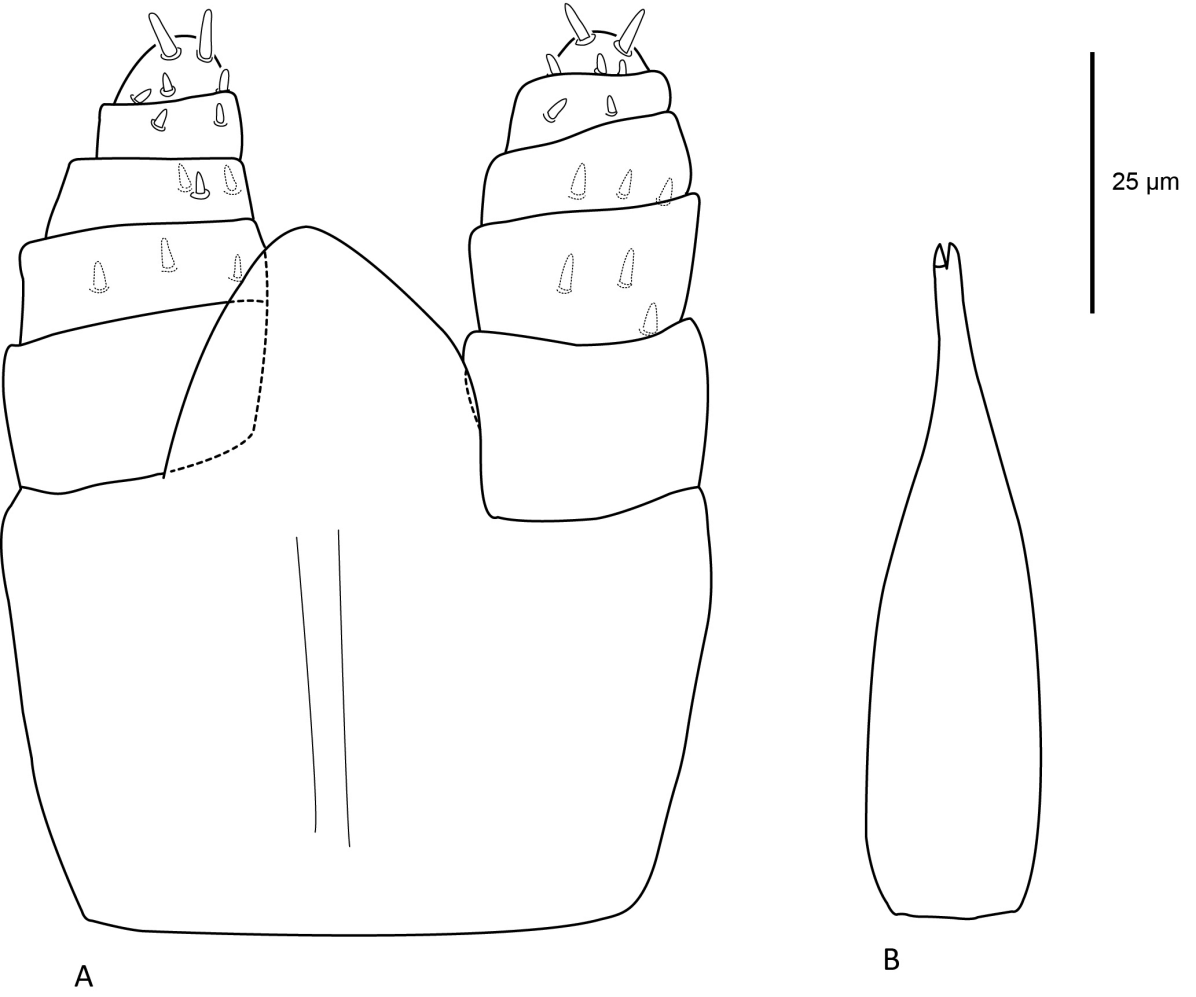
Female *Sternostomagallowayi* sp. n. (A) subcapitulum and palps, ventral aspect; (B) chelicerae.

**Figure 5.**
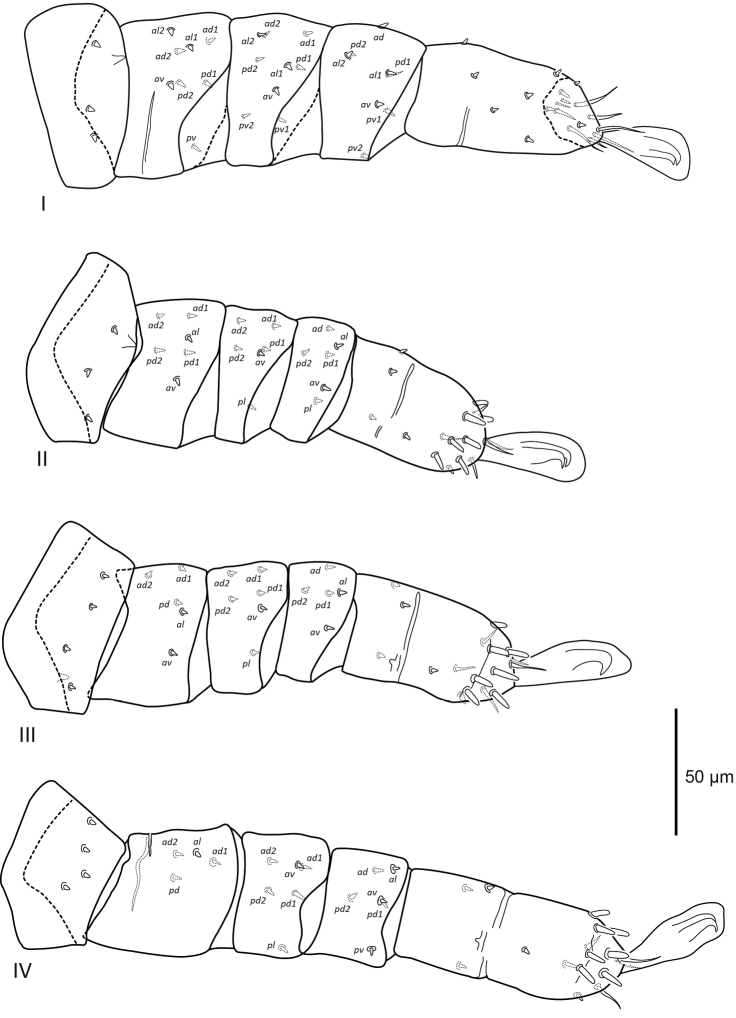
Female *Sternostomagallowayi* sp. n. legs I–IV, coxae omitted.

Figs 1 , 2 , 3 , 4 , 5

##### Material examined.

***Type material*.** Holotype: female (CNC535681) from horned lark (WK357), *Eremophilaalpestris*, Winnipeg, Manitoba, Canada, 22.x.2011, coll: T.D. Galloway. Paratypes: female (CNC991940) same collection information as holotype. Two females (CNC991941, CNC991942) from horned lark (WK625), Winnipeg, Manitoba, Canada, 5.viii.2014, coll. TD Galloway.

**Diagnosis female.** Dorsum with two shields, podosomal shield large, covering most of podosoma with eight pairs of minute setae and two pairs of pore-like structures, opisthosomal shield medium-sized with two pairs of minute setae and four pairs of pore-like structures. Two pairs of minute setae in dorsal opisthosomal unsclerotised integument. Paranal setae on anal shield level with anus, postanal seta absent. Sternal shield longer than wide, three pairs of sternal setae (*st1*–*3*) on shield. Genital shield slightly reticulated lengthwise, broadly rounded posteriorly, seta *st5* on genital shield. Four pairs of minute setae in ventral opisthosomal unsclerotised integument. Ventral subcapitulum without setae. Ventrolateral and apical setae on tarsus II–IV thickened, baculiform and slightly curved distally.

**Description female. *Dorsal idiosoma*** (Figs 1–2). Idiosoma 427 (387–468) long excluding gnathosoma 267 (260–274) wide. Podosomal shield 205 (201–212) long, 202 (193–217) wide covering most of podosoma, with eight pairs of minute setae with rounded tips 1.9 (1.8–2) long in alveoli, and two pairs of pore-like structures in alveoli on shield. Podosomal shield rounded anteriorly, slightly convex lateral margins, posterior margin straight, plate with granular texture and vacuolate areas (Figure 2). Opisthosomal plate quadrangular 150 (148–153) long and 159 (155–161) wide at widest point, slightly wider than long, narrowing posteriorly. Plate with granular texture and vacuolate areas, two pairs of minute setae with rounded tip in alveoli, and four pairs of pore-like structures in alveoli. Dorsal integument finely striated, two pairs of minute setae with rounded tip in unsclerotised integument lateral and posterolateral of opisthosomal shield. Stigmata 11 (9–12) wide, without peritreme, situated near posterior corners of podosomal shield. Anal shield dorsoterminal 60 (56–65) long and 48 (45–51) wide, thickened well sclerotised lateral margins, cribrum present, paranal setae minute with rounded tip 1.8 (1.5–2.1) level with anus, postanal seta absent.

***Ventral idiosoma*** (Figure 3). Sternal shield poorly sclerotised, with weak punctation, slightly wider towards posterior, longer than wide, 124 (120–128) long and 76 (73–79) at widest point, setae *st1* (1.6), *st2* (1.5), *st3* (1.5) in alveoli on shield. Genital shield large, 124 (121–126) long and 76 (73–82) wide level with *st5*, seta *st5* (2.1) on shield, slight reticulations lengthwise, and posterior broadly rounded, pair of lyrifissures *iv5* off genital shield. Cuticle finely striated, four pairs of minute setae with rounded tips in ventral opisthosomal unsclerotised integument.

***Gnathosoma*** (Figure 4). Gnathosoma ventral in position, ventral length including palps 77 (69–82), maximum width 70 (68–73). Deutosternal groove present, deutosternal teeth absent, and subcapitulum without setae. Palps five-segmented, chaetotaxy of palps 0–3–3–2–5, all setae with rounded tips, palp apotele absent. Chelicerae widest proximally and tapering distally, 60 (57–63) long and 19 (18–20) at widest point, with small pointed fixed and moveable digits.

***Legs*** (Figure 5). Excluding ambulacra, length of leg I 257 (226–283), leg II 206 (175–228), leg III 215 (196–243), and leg IV 263 (260–266). Setation of legs I–IV: coxae 2–2–2–1; trochanters 3–3–4–4; femora 8–6–5–4; genua 9–6–6–6; tibiae 8–6–5–6; tarsi 19–17–17–17. Most leg setae simple, minute, with rounded tip, a few apical tarsal setae filamentous. Ventrolateral and apical setae on tarsus II–IV (7.5) thickened, baculiform and slightly curved distally.

***Male and immatures***. unknown

##### Etymology.

This species is named after Dr. Terry D Galloway, who has tirelessly collected nasal mites and other bird-associated arthropods for many years, and has given me the opportunity to continue my studies of these unique mites.

**Remarks**. The female of *Sternostomagallowayi* sp. n. is most similar to those of *S.sialiphilus* Hyland and Ford, and *S.loxiae* Fain. These species each have two dorsal shields which are similar in extent and shape, enlarged and elongated ventrolateral and apical setae on tarsus II–IV, no setae in the unsclerotised dorsal podosomal integument, four pairs of setae in the ventral opisthosomal integument, two or less pairs of setae in the dorsal opisthosomal integument, and lack a postanal seta. *Sternostomasialiphilus* has been collected from the bank swallow (*Ripariariparia*) in Canada, and the eastern bluebird (*Sialiasialis*) in the United States (Hyland and Ford 1961, Knee et al. 2008). *Sternostomaloxiae* has been collected from the red crossbill (*Loxiacurvirostra*) in Canada and Belgium, from the American yellow warbler (*Dendroicapetechia*), and mountain bluebird (*Sialiacurrucoides*) in Canada (Fain 1965, Knee et al. 2008, Knee and Galloway 2017).

Female *S.gallowayi* can be distinguished from that of *S.sialiphilus* and *S.loxiae* by having eight pairs of setae and two pairs of pores on the podosomal shield, *S.sialiphilus* has nine pairs of setae, *S.loxiae* has seven pairs of setae; two pairs of setae and four pairs of pores on the opisthosomal shield, *S.sialiphilus* has one pair of setae and three pairs of pores, *S.loxiae* has three pairs of setae and two pairs of pores on the shield; two pairs of setae in the dorsal opisthosomal unsclerotised integument, *S.sialiphilus* and *S.loxiae* have one pair; paranal setae level with anus, *S.sialiphilus* and *S.loxiae* the paranal setae are posterior to the anus; baculiform ventrolateral and apical setae on tarsus II–IV which are not distally inflated, *S.sialiphilus* and *S.loxiae* have distally inflated ventrolateral and apical setae on tarsus II–IV. *Sternostomagallowayi* differs specifically from *S.sialiphilus* by the presence of seta *st5* on the genital shield, which is absent in *S.sialiphilus*, and the absence of contiguous alveoli between the podosomal and opisthosomal shields which are present in *S.sialiphilus*. *Sternostomagallowayi* differs specifically from *S.loxiae* by having slight reticulation lengthwise on the genital shield, this reticulation is well-developed in *S.loxiae* (Fain 1966a). Comparisons were made using species descriptions from the literature and examining voucher material.

Horned larks are not commonly submitted to wildlife rehabilitation hospitals in Manitoba. Only six specimens have been submitted since 1994, five of which were examined for nasal mites. Of these, two were infested with *S.gallowayi*.

#### Genus Vitznyssus

Taxon classificationAnimaliaMesostigmataRhinonyssidae

Castro, 1948

##### Type species:

*Dermanyssusnitschi* Giebel, 1871 (=*Vitznyssuscaprimulgi* (Fain, 1957))

##### Diagnosis.

Female mites of *Vitznyssus* species are defined by Butenko (1984) as relatively long and slim, with long and thin legs, stigma with short peritreme level with coxa III, anal shield with well-defined cribrum, single podosomal shield on dorsal idiosoma, chelicerae long and thin distally with thickened bases, well-developed tritosternum and deutosternum with denticles.

##### Remarks.

*Vitznyssus* is a small genus comprised of seven species collected from two disparate orders of birds: *V.afrotis* (Fain), *V.neotis* (Fain), *V.tetragis* Butenko, *V.vitzthumi* (Fain) from Otididae, Gruiformes; *V.caprimulgi* (Fain), *V.scotornis* (Fain), and *V.tsachevi* Dimov and Rojas from Caprimulgidae, Caprimulgiformes (Fain 1957b, 1966b, Butenko 1984, Dimov and de Rojas 2012). Butenko (1984) placed five *Astridiella* Fain, 1957 species described by Fain into *Vitznyssus*.

#### Vitznyssus erici sp. n.

Taxon classificationAnimaliaMesostigmataRhinonyssidae

http://zoobank.org/FF7BF246-7432-4B04-B887-0AFA708B1082

Figs 6 , 7 , 8 , 9

##### Material examined.

***Type material*.** Holotype: female (CNC535647) from common nighthawk (WK273), *Chordeilesminor*, Winnipeg, Manitoba, Canada, 4.ix.2010, coll: T.D. Galloway. Paratypes: two females (CNC991938, CNC991939) same collection information as holotype.

##### Diagnosis female.

Large mite with one dorsal shield, podosomal shield longer than wide, rounded anteriorly, constricted posteriorly with irregular margins posterolaterally, six pairs of setiform setae, vacuolate areas and irregular transverse lines on podosomal shield. Subposterior setal pair on podosomal shield elongate, nearly twice as long as all other podosomal shield setae. Four pairs of setiform setae lateral and posterolateral of podosomal shield. Doral and ventral hysterosoma without small shieldlets. Sternal shield small, poorly sclerotised, constricted posteriorly, seta *st1* and lyrifissure *iv1* on sternal shield, setae *st2*, *st3*, and lyrifissure *iv2* off sternal shield. Genital shield elongate and narrow with parallel sides, seta *st5* off genital shield. Paranal setae setiform with filamentous tip anterior to anus, postanal seta setiform with filamentous tip slightly shorter than paranal setae. Well-developed tritosternum with paired laciniae. Palp apotele two-tined with bifid tips.

##### Description female.

**Figure 6.**
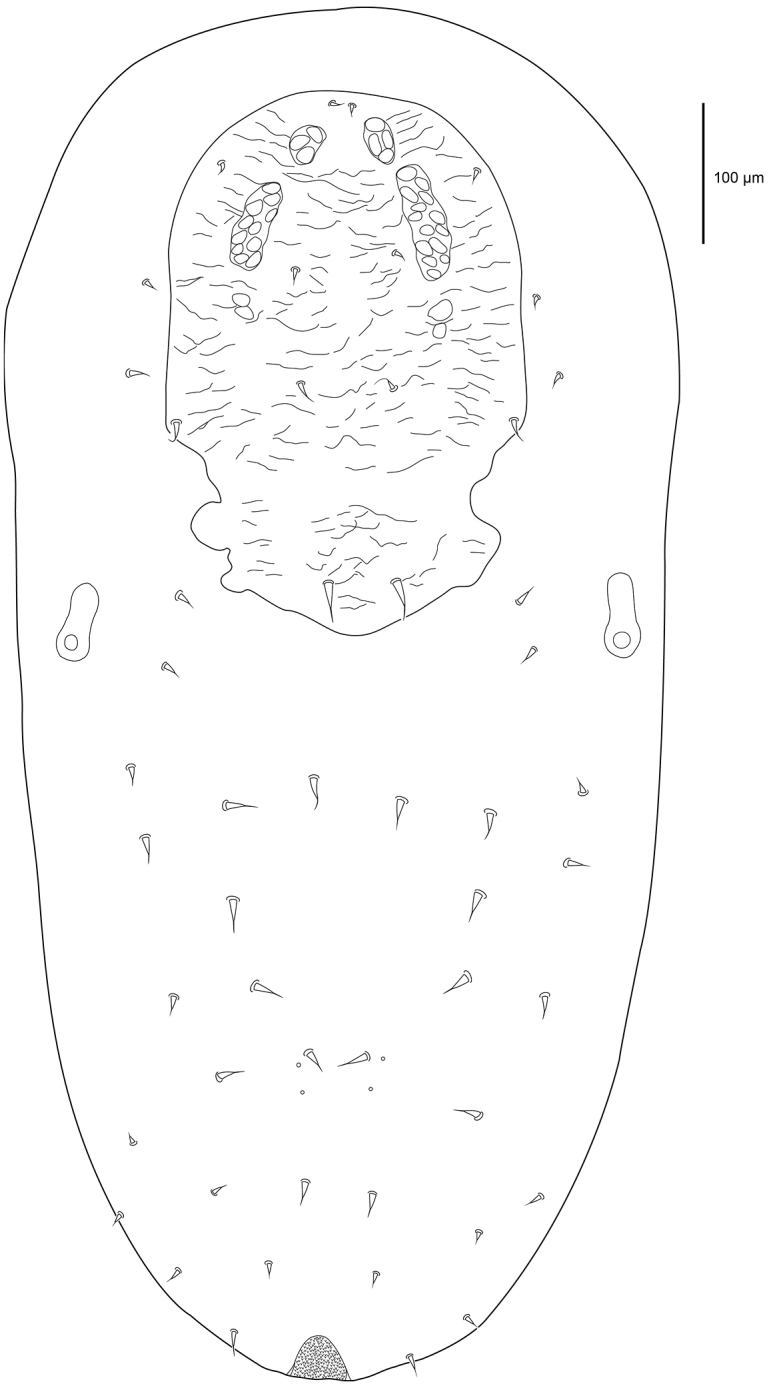
Female *Vitznyssuserici* sp. n. dorsal idiosoma.

***Dorsal idiosoma*** (Figure 6). Idiosoma 1016 (953–1168) long excluding gnathosoma, 493 (434–550) wide. Podosomal shield 387 (372–410) long, 261 (256–270) wide, rounded anteriorly, constricted posteriorly with irregular margins posterolaterally, six pairs of setiform setae, vacuolate areas and irregular transverse lines on podosomal shield. Subposterior setal pair on podosomal shield elongate 23 (21–27), almost twice as long as all other podosomal shield setae 12 (8–16). Dorsal integument finely striated, four pairs of setiform setae in unsclerotised integument lateral and posterolateral of podosomal shield, 15–16 pairs of setiform setae 20 (15–22) and two pairs of pore-like structures in dorsal opisthosomal unsclerotised integument. Stigmata with short peritremes 54 (53–55).

**Figure 7.**
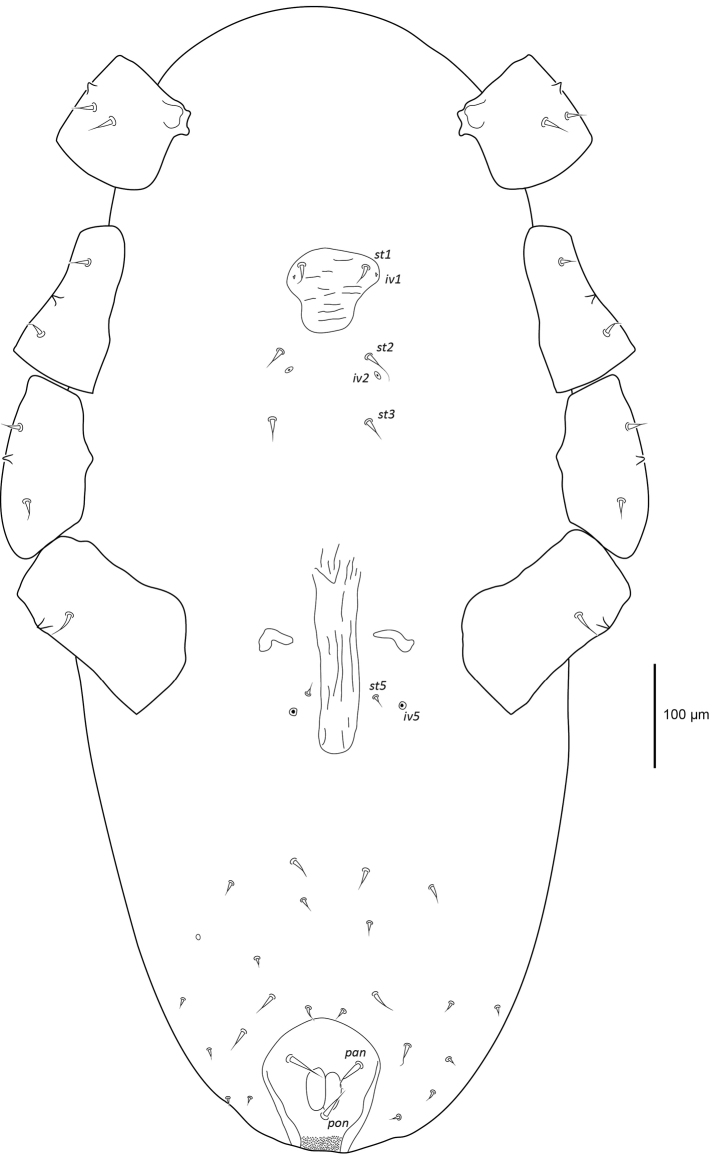
Female *Vitznyssuserici* sp. n. ventral idiosoma including coxae.

***Ventral idiosoma*** (Figure 7). Sternal shield reduced and poorly sclerotised, 83 (80–87) long and 86 (86–87) maximum width anteriorly, constricted posteriorly, shield with irregular transverse lines, seta *st1* (18) and lyrifissure *iv1* on sternal shield. Setae *st2* (20), *st3* (19), and lyrifissure *iv2* in unsclerotised integument off sternal shield. Genital shield elongate and narrow, 196 (193–200) long and 40 (37–43) wide, parallel sides and reticulated lengthwise, seta *st5* (11) and pair of lyrifissures *iv5* off genital shield. Cuticle finely striated, 11 pairs of setiform setae in ventral opisthosomal unsclerotised integument. Anal shield 162 (155–169) long and 114 (112–115) wide, thickened lateral margins, cribrum present, paranal setae 46 (45–48) setiform with filamentous tip anterior to anus, postanal seta 32 (30–36) setiform with filamentous tip slightly shorter than paranal setae.

**Figure 8.**
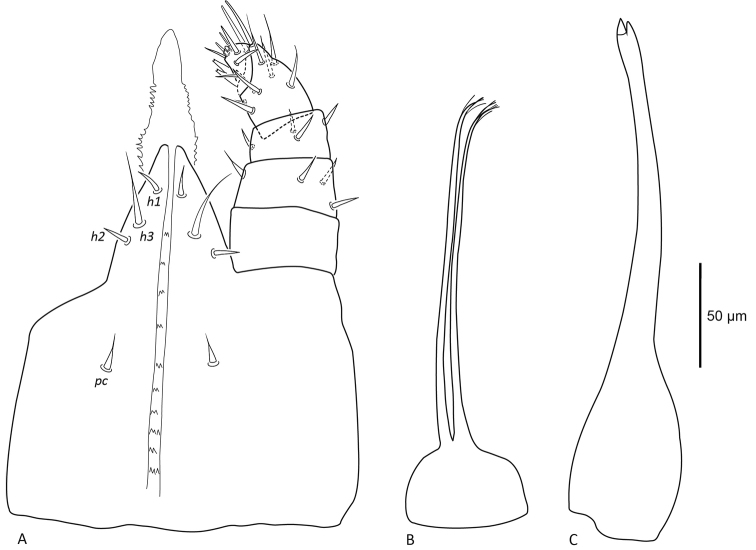
Female *Vitznyssuserici* sp. n. (A) subcapitulum and palp, ventral aspect; (B) tritosternum; (C) chelicerae.

***Gnathosoma*** (Figure 8). Gnathosoma terminal, ventral length including palps 236 (225–242), width 160 (152–166) posterior to *pc* seta. Subcapitulum with 10 rows of paired deutosternal denticles. Subcapitular setae setiform, *pc* 20 (15–23), *h1* 13 (12–14), *h2* 13 (11–15), and *h3* 33 (26–39). Well-developed tritosternum 233 long, with paired laciniae. Palps five-segmented, chaetotaxy of palps 0–4–4–8–9, palp apotele two-tined with bifid tips. Chelicerae elongate 242 (237–249), expanded proximally 50 (45–56), marked constriction distally with small pointed moveable and fixed digits.

**Figure 9.**
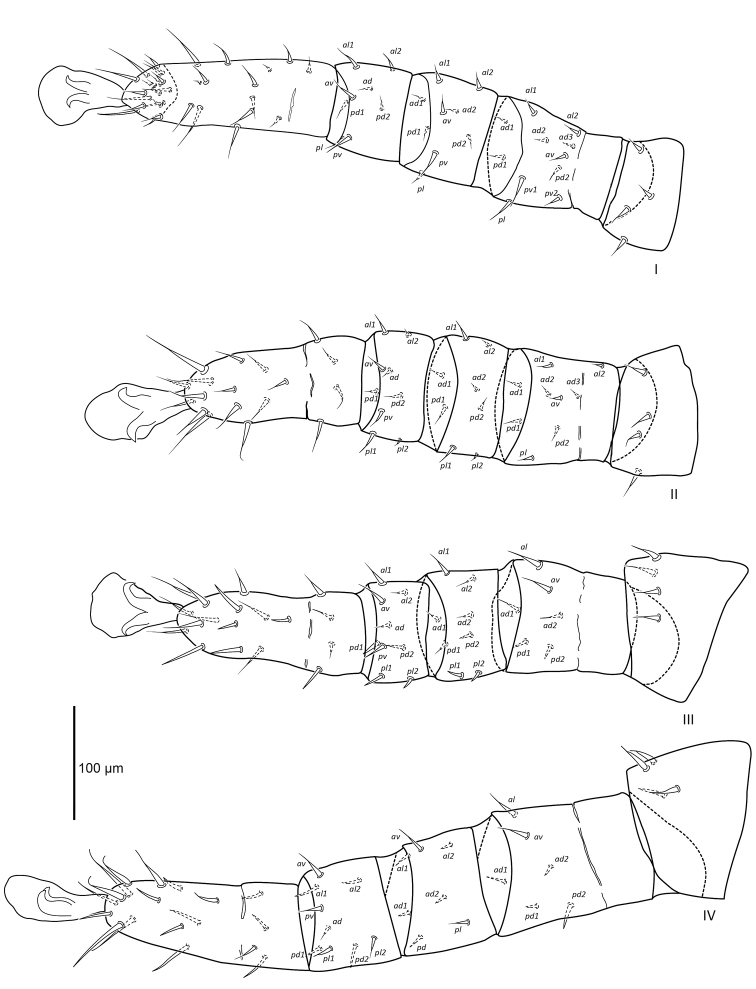
Female *Vitznyssuserici* sp. n. legs I–IV, coxae omitted.

***Legs*** (Figure 9). Excluding ambulacra, length of leg I 541 (483–591), leg II 520 (487–541), leg III 518 (512–521), and leg IV 615 (596–627). Setation of legs I–IV: coxae 2–2–2–1; trochanters 4–4–4–4; femora I (2–3/1,2/2–1), II (2–3/1,2/0–1), III (1–2/1,2/0–0), IV (1–2/1,2/0–0); genua I (2–2/1,2/1–1), II (2–2/0,2/0–2), III (2–2/0,2/0–2), IV (2–2/1,1/0–1); tibiae I (2–1/1,2/1–1), II (2–1/1,2/1–2), III (2–1/1,2/1–2), IV (2–1/1,2/1–2); tarsi 33–18–18–18. Leg setae are setiform with filamentous tip.

***Male and immatures***. Unknown.

##### Etymology.

This species is named after Eric Shewchuk, a close friend who has been beside me throughout the entirety of my studies on mites.

##### Remarks.

The female of *Vitznyssuserici* sp. n. is most similar that of *V.afrotis* Fain, which was described from the southern black bustard (*Afrotisafra*) in South Africa (Fain 1966b). These two species are most similar in that they are large mites (at least 1000 µm long), and both have: one dorsal shield which is longer than wide and eroded along the margins, especially posterolaterally; sternal shield reduced, with seta *st1* on the shield; elongate and narrow genital shield; palp apotele two-tined with bifid tips; elongate tritosternum with two laciniae; elongate paranal setae anterior to anus, and elongate postanal setae present.

Female *V.erici* differ from that of *V.afrotis* by: the presence of irregular transverse lines on the podosomal shield, which are absent in *V.afrotis*; the posterolateral margins of the podosomal shield are more eroded, invaginated and irregular than that of *V.afrotis*; the anterior margin of the podosomal shield is broadly rounded with no setae in the integument anterior of the shield, the anterior margin is slightly eroded in *V.afrotis* and a pair of setae are off the shield anteriorly; the sternal shield is larger, with a posteromedial projection, and lyrifissure *iv1* is on the shield, *V.afrotis* sternal shield is smaller and without a posteromedial projection, *iv1* is off the shield; the genital shield margins are parallel and not flared posteriorly like they are in *V.afrotis*; and by the absence of small accessory shieldlets on the dorsal or ventral hysterosoma, which are present in *V.afrotis*. Fain (1966b) described *V.afrotis* as lacking a sternal shield; however, the holotype and paratype specimens examined have a small narrow sternal shield with seta *st1* on the shield. Comparisons were made using the species description for *V.afrotis* and examination of the holotype and paratype specimens loaned from the Royal Belgian Institute for Natural Sciences.

Including *V.erici* there are now four *Vitznyssus* species known from Otididae (bustards) and four species known from Caprimulgidae (nightjars) hosts. Bustards only occur in the Eastern Hemisphere, while nightjars are broadly distributed in both hemispheres. Considering the geographic distribution of these mites and the disparate host bird orders, the monophyly of the genus and species boundaries should be investigated using molecular markers and morphometric analyses.

The common nighthawk (*Chordeilesminor*) has been a focus for studies of ectoparasites in Manitoba (Galloway 2006, Galloway and Lamb 2015). Despite the apparent decline in populations of common nighthawk in the province (Taylor 1996), these birds are frequently submitted to rehabilitation hospitals. However, using the methods described here, nasal mites are either rare or rarely collected. Out of 138 common nighthawks examined for this study since 1999, nasal mites were collected from only one. The conservation status of this mite certainly deserves consideration.

## Supplementary Material

XML Treatment for Sternostoma

XML Treatment for Sternostoma gallowayi

XML Treatment for Vitznyssus

XML Treatment for Vitznyssus erici
